# HIF1α-dependent glycolysis promotes macrophage functional activities in protecting against bacterial and fungal infection

**DOI:** 10.1038/s41598-018-22039-9

**Published:** 2018-02-26

**Authors:** Chunxiao Li, Yu Wang, Yan Li, Qing Yu, Xi Jin, Xiao Wang, Anna Jia, Ying Hu, Linian Han, Jian Wang, Hui Yang, Dapeng Yan, Yujing Bi, Guangwei Liu

**Affiliations:** 1 Department of Immunology, School of Basic Medical Sciences, Fudan University, Shanghai, 200032 China; 2 Key Laboratory of Cell Proliferation and Regulation Biology of Ministry of Education, Institute of Cell Biology, College of Life Sciences, Beijing Normal University, Beijing, 100875 China; 3 State Key Laboratory of Pathogen and Biosecurity, Beijing Institute of Microbiology and Epidemiology, Beijing, 100071 China

## Abstract

Macrophages are important innate immune defense system cells in the fight against bacterial and fungal pathogenic infections. They exhibit significant plasticity, particularly with their ability to undergo functional differentiation. Additionally, HIF1α is critically involved in the functional differentiation of macrophages during inflammation. However, the role of macrophage HIF1α in protecting against different pathogenic infections remains unclear. In this study, we investigated and compared the roles of HIF1α in different macrophage functional effects of bacterial and fungal infections *in vitro* and *in vivo*. We found that bacterial and fungal infections produced similar effects on macrophage functional differentiation. HIF1α deficiency inhibited pro-inflammatory macrophage functional activities when cells were stimulated with LPS or curdlan *in vitro* or when mice were infected with *L. monocytogenes* or *C. albicans in vivo*, thus decreasing pro-inflammatory TNFα and IL-6 secretion associated with pathogenic microorganism survival. Alteration of glycolytic pathway activation was required for the functional differentiation of pro-inflammatory macrophages in protecting against bacterial and fungal infections. Thus, the HIF1α-dependent glycolytic pathway is essential for pro-inflammatory macrophage functional differentiation in protecting against bacterial and fungal infections.

## Introduction

Macrophages are the important innate immune defense system cell against bacteria, viruses and fungal pathogen infections, and they exhibit significant plasticity, particularly with their ability to undergo functional differentiation between functional phenotypes, depending on microenvironments^1–4^. Macrophages activated by Toll-like receptor (TLR) ligands, such as LPS or/and IFNγ, are called pro-inflammatory macrophages (classically activated macrophages) and produce a large amount of pro-inflammatory cytokines, including TNFα, and increased amounts of nitric oxide (NO) through the enhanced expression of inducible NO synthase (iNOS) and are critical in eradicating pathogenic microorganisms^5–8^. In contrast, macrophages activated with IL-4 or IL-13, are called anti-inflammatory macrophages (alternatively activated macrophages) and are characterized by the production of a large amount of anti-inflammatory cytokines, including IL-10, and a high expression of arginase 1 (Arg1), chitinase 3-like 3 protein (Ym1) and are particularly important in protecting against parasitic infections^9–12^. Although the differentiation of macrophages is widely studied^4,11,13,14^, the macrophage functional activation effects and mechanisms of different kinds of pathogenic microorganisms (especially fungal infections) remain unclear.

Hypoxia inducible factor 1 (HIF1) is a heterodimeric transcription complex consisting of two subunits, HIF1α and HIF1β^15,16^. HIF1α is the oxygen-regulated subunit, which rapidly accumulates in cells exposed to hypoxia and has been shown to be a critical transcriptional factor in controlling innate immune function^17^. Hypoxia is a physiological environment created by the host innate immune system in its defense against pathogens^18^. Our previous work showed that pro-inflammatory cytokines or pathogenic microorganisms can induce Myc-dependent and HIF1α-dependent transcriptional programs that regulate the robust bioenergetic support required for an inflammatory response while inhibiting Myc-dependent proliferation^19^. However, the significance of HIF1α-glycolytic pathway signals in protecting against different kinds of pathogenic microorganism infections remains unclear.

In this study, we investigated and compared the different macrophage functional effects of bacterial and fungal infections *in vitro* and *in vivo*. We found that bacterial and fungal infections exhibited similar effects on macrophage phenotypic differentiation. HIF1α deficiency inhibits pro-inflammatory macrophage functional differentiation in cells stimulated with LPS or curdlan^20,21^ (a homopolymer of D-glucose and the major cellular component of fungal extracts of *C. albicans*) *in vitro* or in mice infected with *Listeria monocytogenes* (*L. monocytogenes*) or *C. albicans in vivo* by decreasing pro-inflammatory TNFα and IL-6 secretions associated with pathogenic microorganism survival. Alteration of glycolytic pathway activation was required for the differentiation of pro-inflammatory macrophages needed in protecting against bacterial and fungal infections, which should contribute to our understanding of targeted therapy in protecting against different pathogenic microorganism infections in clinical settings.

## Materials and Methods

### Mice

All animal experiments were performed in accordance with protocols approved by the Animal Ethics Committee of Fudan University (Shanghai, China) or (CLS-EAW-2017-003) Beijing Normal University (Beijing, China). *Hif1α*^flox/flox^ and *Lyz*-*Cre* mice were obtained from The Jackson Laboratory (Bar Harbor, ME, USA). C57BL/6 mice were obtained from the Fudan University Experimental Animal Center (Shanghai, China) or the Weitonglihua Experimental Animal Limited Company (Beijing, China). All mice had been backcrossed to the age of 6–12 weeks. WT control mice were of the same genetic background and, where relevant, included *Cre*^+^ mice to account for the effects of Cre (no adverse effects due to Cre expression itself were observed *in vitro* or *in vivo*).

### Bacterial and fungal infection

For bacterial infections, mice were injected intravenously with 1 × 10^5^ CFU of *L. monocytogenes*. At 48 h, mice were killed for analysis, as described previously^16^. Infected liver or spleen samples were fixed in 4% paraformaldehyde, embedded in paraffin and stained with H&E. Spleens were teased with a 32 G needle using RPMI 1640 containing 5% FBS in a petri dish and digested in RPMI 1640 containing collagenase D (400 U/ml; Roche) in a shaking water bath at 37 °C for 35 min. Subsequently, 0.5 ml of 0.1 M EDTA was added, and samples were incubated on ice for 5 min and then transferred to a 50 ml tube by filtering through a 70 µm cell strainer. After a centrifugation step, the supernatant was removed and splenic cells were stained with fluorescent antibodies and macrophages (CD11b^+^, F4/80^+^, TCR^−^, CD19^−^, NK1.1^−^, Ly6G^−^, and 7-AAD^−^) were sorted on a FACSAria II (Becton Dickinson). Peritoneal cells were harvested from mouse peritoneal cavities by lavage and the peritoneal exudate macrophages (PEMs; 7AAD^−^, CD11b^+^, and F4/80^+^) were sorted on a MoFlo XDP (Beckman Coulter). The mouse macrophages were stimulated with LPS (100 ng/mL) for 6 h for subsequent intracellular staining, ELISA, or RNA isolation. For fungal infections, mice were injected intravenously with 2 × 10^5^ of live *C. albicans* yeast *in vivo*. After 9 days, mice were killed for analysis. Infected kidney samples were fixed in 4% paraformaldehyde, embedded in paraffin, and stained with H&E. Infected kidney and/or liver samples were collected and CFU counts were determined. Splenocytes were harvested and stimulated with 10^6^ of heat-killed *C. albicans* per ml of cells for 48 h *in vitro* for subsequent intracellular staining, ELISA, or RNA isolation. Under some conditions, macrophages are isolated from the indicated mice and after an infection *in vitro* with *L. monocytogenes* and *C. albicans* yeast for 10–12 h, we performed intracellular staining, ELISA, RNA isolation, or functional analyses. For drug treatments, cells were incubated either with vehicle, CoCl_2_ (200 µM; Calbiochem), 2-ME (2 µM; Calbiochem), or 2-DG (1 mmol/L; Sigma) for 0.5–1 h before stimulation.

### Measurement of bacterial and fungal burdens

On the indicated days after challenge infections, infected livers and kidneys were harvested and homogenized in PBS. For bacterial infections, serial dilutions of homogenates were plated on LB agar plates and incubated at 37 °C for 24 h. For fungal infections, serial dilutions of homogenates were plated on Sabouraud dextrose agar (SDA) plates and incubated at 37 °C for 48 h. Bacterial and fungal colony forming units (CFU) were subsequently counted.

### Monoclonal antibody (mAb) and flow cytometry assays

For FCM analysis of cell surface markers, cells were stained with antibodies in PBS containing 0.1% (wt/vol) BSA and 0.1% NaN_3_, as described previously^22^. Anti-CD11b (M1/70), anti-F4/80 (BM8), anti-CD14 (61D3), and anti-CD68 (FA11) were obtained from eBioscience. Anti-CD45 (TU116) was obtained from BD Biosciences. Anti-CD3 (145-2C11) and anti-CD19 (6D5) were obtained from Miltenyi Biotec. For intracellular staining, TNFα expression (MP6-XT22; Biolegend) was analyzed by flow cytometry according to the manufacturer’s instructions. For TNFα expression analysis, cells isolated from the indicated organs were restimulated with LPS (L2630; Sigma-Aldrich, St. Louis, MO, USA) for 5 h for the CD11b^+^ F4/80^+^ macrophage analysis, while GolgiStop (554724; BD Biosciences) was added for the last 2 h of incubation. After surface staining and washing, the cells were fixed immediately with Cytofix/Cytoperm solution (554714; BD Biosciences) and were stained with anti-TNF-α antibody (MP6-XT22) purchased from eBioscience. Flow cytometry data were acquired on a FACSCalibur instrument (Becton Dickinson, CA, USA), and data were analyzed with FlowJo (Tree Star, San Carlos, CA, USA).

### Glycolysis flux assay

Glycolysis in macrophages was determined by measuring the detritiation of [3-^3^H]-glucose as described previously^23^. In brief, the assay was initiated by adding 1 μCi of [3-^3^H]-glucose (Perkin Elmer), and 2 h later, medium was transferred to microcentrifuge tubes containing 50 μl 5 N HCL. The microcentrifuge tubes were then placed in 20 ml scintillation vials containing 0.5 ml water, and the vials were capped and sealed. ^3^H_2_O was separated from un-metabolized [3-^3^H]-glucose by evaporation diffusion for 24 h at room temperature. Cell-free samples containing 1 μCi of ^3^H-glucose were included as a background control.

### NO production

For NO production, culture supernatant was incubated with the Griess reagent (G4410; Sigma-Aldrich), as described previously^24^.

### Quantitative RT-PCR and Immunoblot

RNA was extracted with an RNeasy kit (Qiagen, Dusseldorf, Germany), and cDNA was synthesized using SuperScript III reverse transcriptase (Invitrogen, Carlsbad, CA, USA). An ABI 7900 Real-time PCR system was used for quantitative PCR, with primer and probe sets obtained from Applied Biosystems. The results were analyzed using SDS 2.1 software (Applied Biosystems). The cycling threshold value of the endogenous control gene (Hprt1, encoding hypoxanthine guanine phosphoribosyl transferase) was subtracted from the cycling threshold. The expression of each target gene is presented as the ‘fold change’ relative to that of control samples, as described previously^25^. Immunolbot was performed with the following antibodies: HIF1α (R&D system) and β-actin (Sigma), as described previously^16^.

### Human macrophage functions

For assays of human macrophage functional activities, CD14^+^ monocytes from healthy human donors (2W-400C; Lonza) were cultured, and monocytes-derived macrophages were differentiated with rHu M-CSF (25 ng/mL; R&D system) for 7 days. Subsequently, live macrophages (7-AAD^−^ and CD11b^+^) were sorted with a MoFlo XDP (Beckman Coulter) and then stimulated with LPS for mRNA analysis or ELISAs.

### Statistical analyses

All data are presented as the means ± SD. The non-parametric U test was applied for the comparison of means and was used to compare differences between groups. Comparison of the survival curves was performed using the Log-Rank (Mantel-Cox) test. A *P* value (alpha-value) of less than 0.05 was considered to be statistically significant.

## Results

### HIF1α-dependent glycolysis is associated with pro-inflammatory macrophage differentiation

**Figure 1:**
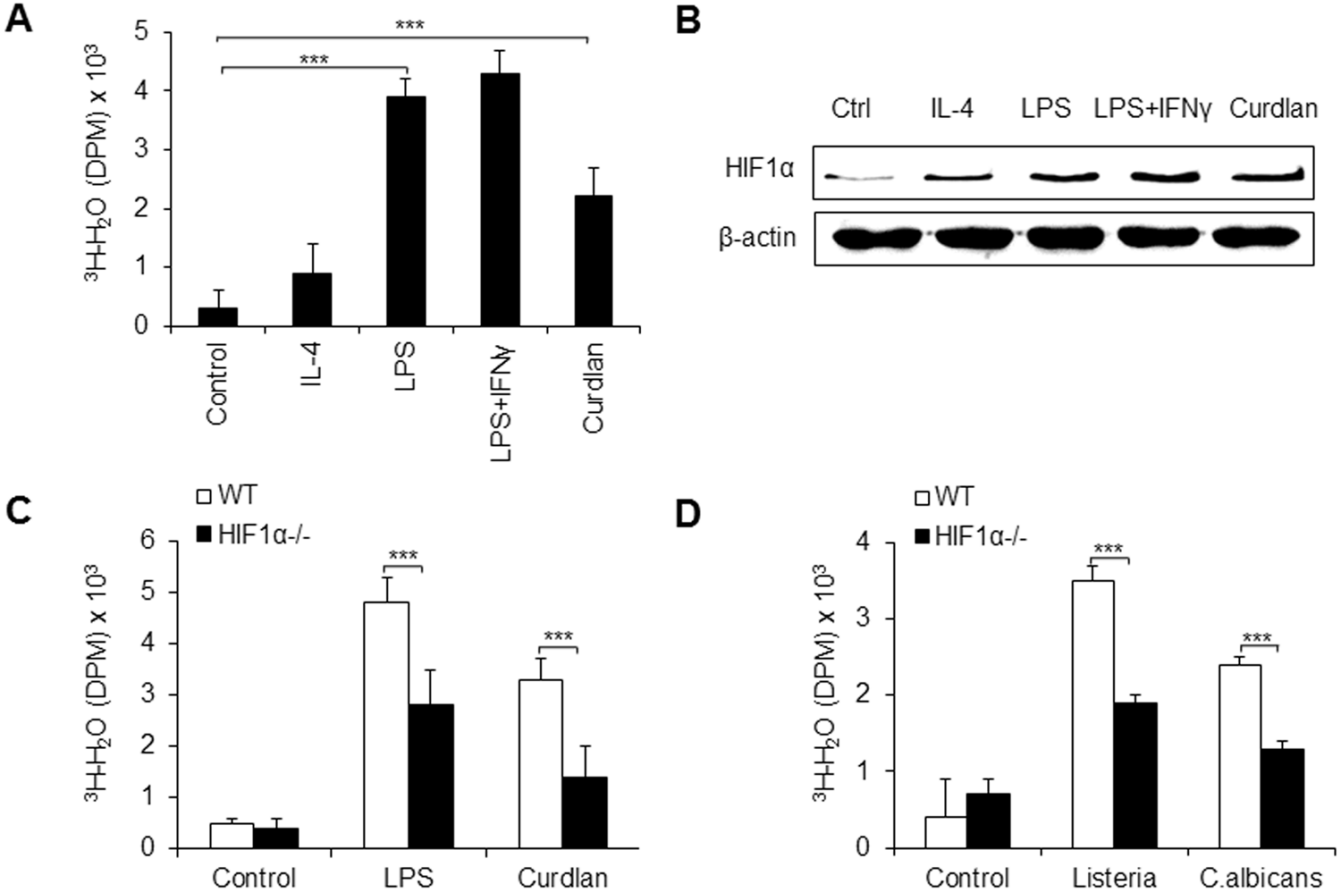
HIF1α-dependent glycolysis is associated with pro-inflammatory macrophage differentiation during inflammation. Sorted peritoneal exudate macrophages (PEMs) from C57BL/6 mice were stimulated with IL-4 (1000 U), LPS (100 ng/mL), LPS + IFNγ (100 ng/mL) or curdlan (100 ng/mL) for 10–12 h, and the glycolytic pathway activity was determined by the generation of ^3^H-labeled H_2_O from [3-^3^H]-glucose (**A**). HIF1α protein expression was determined using immunoblot (**B**). Sorted PEMs from wild-type (WT) C57BL/6 mice or *Hif1α*^flox/flox^; *Lyz*-*Cre* (HIF1α^−/−^) mice were stimulated with the indicated stimuli for 10–12 h, and the glycolytic pathway activity was determined (**C,D**). Data is presented as the means ± SD (n = 4). One representative experiment of three to four independent experiments is shown. *P < 0.05 and ***P < 0.001, compared with the indicated groups.

The glycolytic activities of macrophages were first investigated using different macrophage polarization conditions. TLR ligands are classically considered to induce pro-inflammatory macrophage differentiation, and IL-4 cytokines are considered to induce anti-inflammatory macrophage differentiation, whereas dectin-1, a c-type lectin specific for β-glucans, induces fungal infection responses^26–28^. Therefore, we compared the *in vitro* adjuvant activity on macrophages of lipopolysaccharides (LPS), a widely used ligand for TLR4, IL-4, and curdlan, a prototypic agonist for dectin-1, and evaluated the macrophages’ glycolytic pathway activity alterations. The glycolytic pathway activity of differentiated macrophages was measured by the generation of ^3^H-H_2_O from [3-^3^H]-glucose. Sorted PEMs were stimulated and the glycolytic pathway activity was determined. As reported previously^29–31^, pro-inflammatory macrophage-inducing factor LPS or LPS + IFNγ significantly increased macrophage glycolytic pathway activity, while anti-inflammatory macrophage-inducing factor IL-4 did not significantly alter their glycolytic pathway activity. However, curdlan significantly increased the glycolytic pathway activity of macrophages, but its effect was weaker than that of LPS or LPS + IFNγ treatment groups (Figs 1A and S1). These data showed that fungal infection likely induces weaker glycolytic pathway activity than that of pro-inflammatory macrophage differentiation. As previously reported^32,33^, HIF1α is critically involved in the modulation of glycolytic pathway activity. We then determined the expression of HIF1α in cells. Consistently, HIF1α expression was higher during pro-inflammatory macrophage inducing conditions and with curdlan stimulation, but not during anti-inflammatory macrophage inducing conditions (Figs 1B and S2). These data showed that HIF1α-dependent glycolytic modulation is probably involved in the polarization of macrophages during different kinds of pathogen infections. We next determined whether HIF1α contributes to the alteration of glycolytic pathway activity in differentiated macrophages during different pathogen infections. We used mice with conditional HIF1α deletions. HIF1α^flox/flox^ mice were crossed with LysM-Cre^+/+^ mice, and as a result, HIF1α is specifically deleted in macrophages (called HIF1α^−/−^ hereafter, Fig. S3). As expected, HIF1α deficiency significantly decreased glycolytic pathway activity of differentiated macrophages stimulated with either LPS or curdlan, and this was also observed in cells challenged *in vitro* with *L. monocytogenes* or *C. albicans* (Fig. 1C,D). Taken together, these data suggested that HIF1α-dependent glycolytic pathway activity is associated with macrophage polarization induced by different kinds of pathogenic microorganism infections.

### HIF1α is critical for pro-inflammatory macrophage differentiation *in vitro*

**Figure 2:**
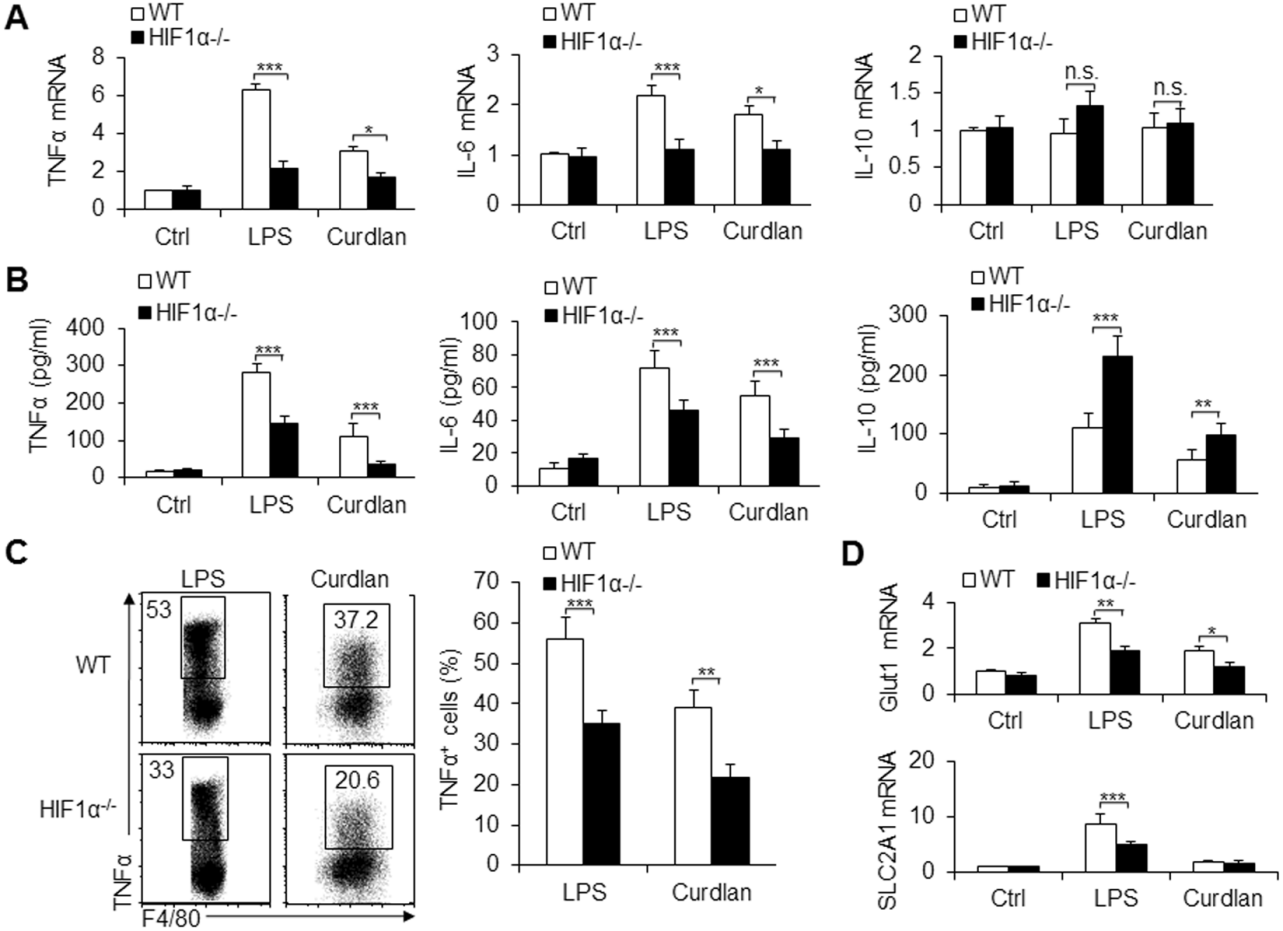
HIF1α is critical for pro-inflammatory macrophage differentiation *in vitro*. Sorted PEMs from WT and HIF1α^−/−^ mice were stimulated with LPS (100 ng/mL) or curdlan (100 ng/mL) for 10–12 h, and the indicated mRNA expression was determined with qPCR (**A**). Supernatant was collected and the concentration of the indicated cytokines was determined with an ELISA (**B**). (**C**) Peritoneal exudate cells from WT and HIF1α^−/−^ mice were activated with the indicated stimuli, and the intracellular expression of TNFα in F4/80^+^ macrophages was determined with flow cytometry; a representative image is shown in the left figure, and data are summarized in the right figure. (**D**) Sorted PEMs were stimulated with LPS or curdlan for 10–12 h, and the mRNA expression of glycolytic pathway associated molecules was determined with qPCR. Data are presented as the means ± SD (n = 3–5). One representative experiment of three independent experiments is shown. *P < 0.05; **P < 0.01 and ***P < 0.001, compared with the indicated groups; n.s. = not significant.

To directly investigate how HIF1α affects macrophage polarization differentiation during different pathogen infections, we observed macrophage functional alterations *in vitro*. In LPS-stimulated cells, HIF1α deficiency significantly inhibited the mRNA expression, secretion and production of the pro-inflammatory cytokine TNFα, IL-6 production, and the production of the anti-inflammatory cytokine IL-10. Since LPS is a classic inducing factor for bacterial infection (Fig. 2A–C), this indicates that HIF1α is required for the differentiation of pro-inflammatory macrophages during bacterial infection. Curdlan, a specific ligand for Dectin-1, which has been shown to consistently induce fungal infection responses, caused similar and weaker pro-inflammatory macrophage differentiation (Figs 2A–C and S4). Glut1 and SLC2A1 have been used as markers of glycolytic pathway activity in macrophages^19^, and HIF1α deficiency significantly blocked these glycolytic molecules’ expression in macrophages (Fig. 2D). This suggested that glycolytic pathway activity is associated with HIF1α-dependent differentiation of pro-inflammatory macrophages.

### HIF1α is required for the differentiation of pro-inflammatory macrophages during bacterial infection

**Figure 3:**
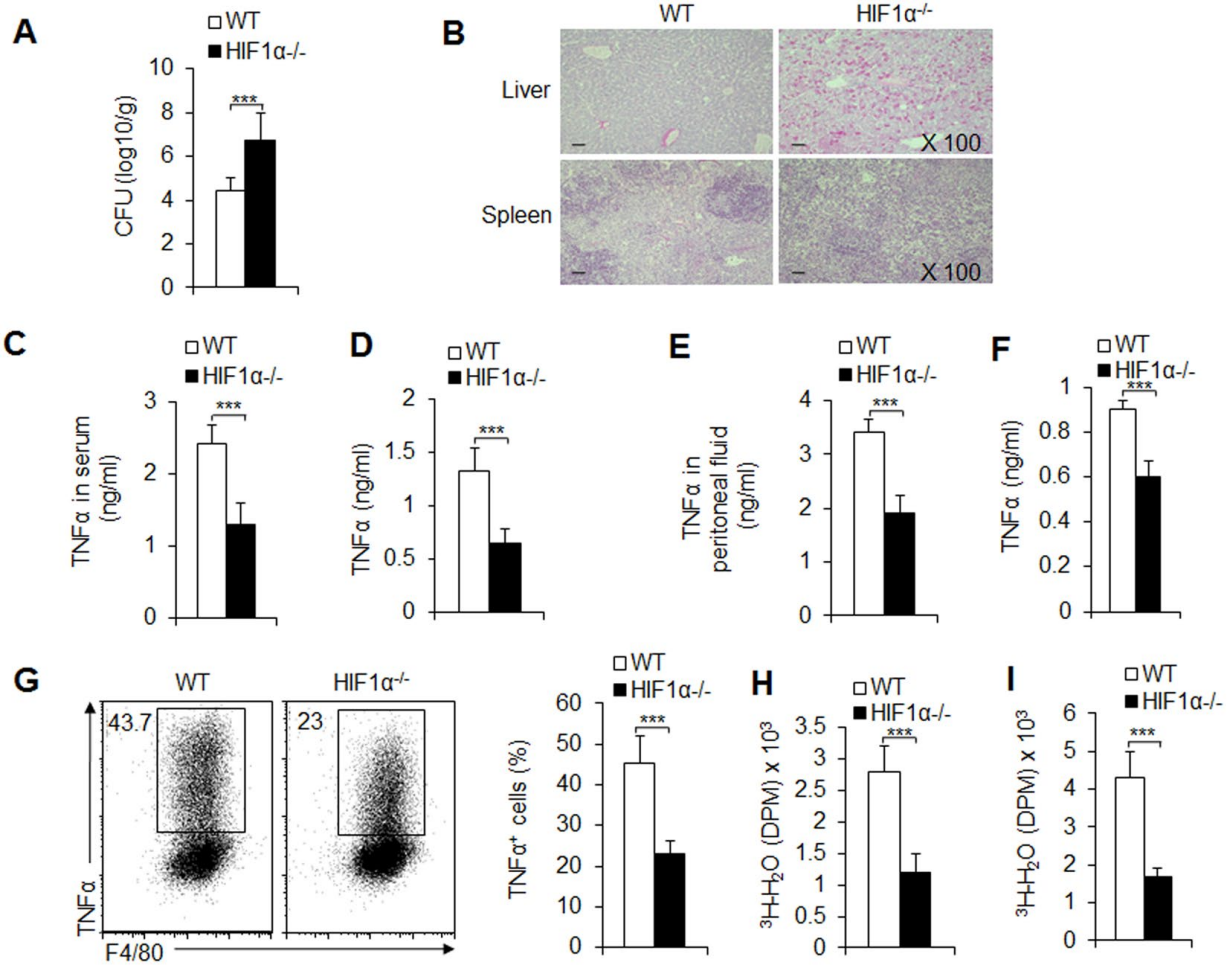
HIF1α is required for pro-inflammatory macrophage differentiation following *Listeria* bacterial infection. C57BL/6 WT or HIF1α^−/−^ mice were i.v. injected with 1 × 10^5^ CFU of *L. monocytogenes* bacteria. 48 h after infection, mouse livers were collected, and the CFU were determined (**A**). Infected mice developed severe infection and inflammatory cell infiltration, as shown by the histological staining of H&E (**B**). At the same time point after infection, serum or peritoneal exudate TNFα levels were determined using an ELISA (**C** and **E**); the sorted splenic macrophages or PEMs were stimulated with LPS for 12 h, the supernatant was collected, and TNFα levels were determined using an ELISA (**D** and **F**); the glycolytic pathway activity of macrophages was also determined (**H** and **I**). TNFα expression in F4/80^+^ macrophages from peritoneal exudates was analyzed with FCM. A representative figure is shown in the left image, and the data are summarized in the right image (**G**). Data are presented as the means ± SD (n = 3–5). One representative experiment of three independent experiments is shown. ***P < 0.001, compared with the indicated groups.

To investigate the significance of HIF1α-dependent differentiation of pro-inflammatory macrophages during different pathogenic microorganism infections, we challenged WT and HIF1α^−/−^ mice with *L. monocytogenes in vivo* at 48 h^34,35^. As shown in the figures, HIF1α^−/−^ mice displayed an earlier onset and a markedly more severe course of infection. The number of *L. monocytogenes* organisms after the challenge showed that the liver had higher bacterial CFU in HIF1α^−/−^ than in the WT controls (Fig. 3A). Microscopic and histological observations revealed a more severe pathological inflammation in the liver and spleen of HIF1α^−/−^ mice (Fig. 3B). Consistently, HIF1α deficiency significantly decreased the level of TNFα in the serum and in peritoneal exudates after *L. monocytogenes* challenge (Fig. 3C and E). Additionally, splenic and peritoneal macrophages showed decreased TNFα secretion and production in HIF1α^−/−^ mice compared with WT control groups (Figs S5A,B and 3D,F and G). NO production, a marker of pro-inflammatory macrophages, is also lower in HIF1α-deficient peritoneal macrophages (Fig. S6). Furthermore, the glycolytic pathway activity of splenic and peritoneal macrophages was significantly decreased in HIF1α^−/−^ genotypes (Figs S5C,D, S7 and 3H,I). Together, these data showed that HIF1α is required for pro-inflammatory macrophage differentiation during bacterial infection.

### HIF1α is required for the differentiation of pro-inflammatory macrophages during fungal infection

**Figure 4:**
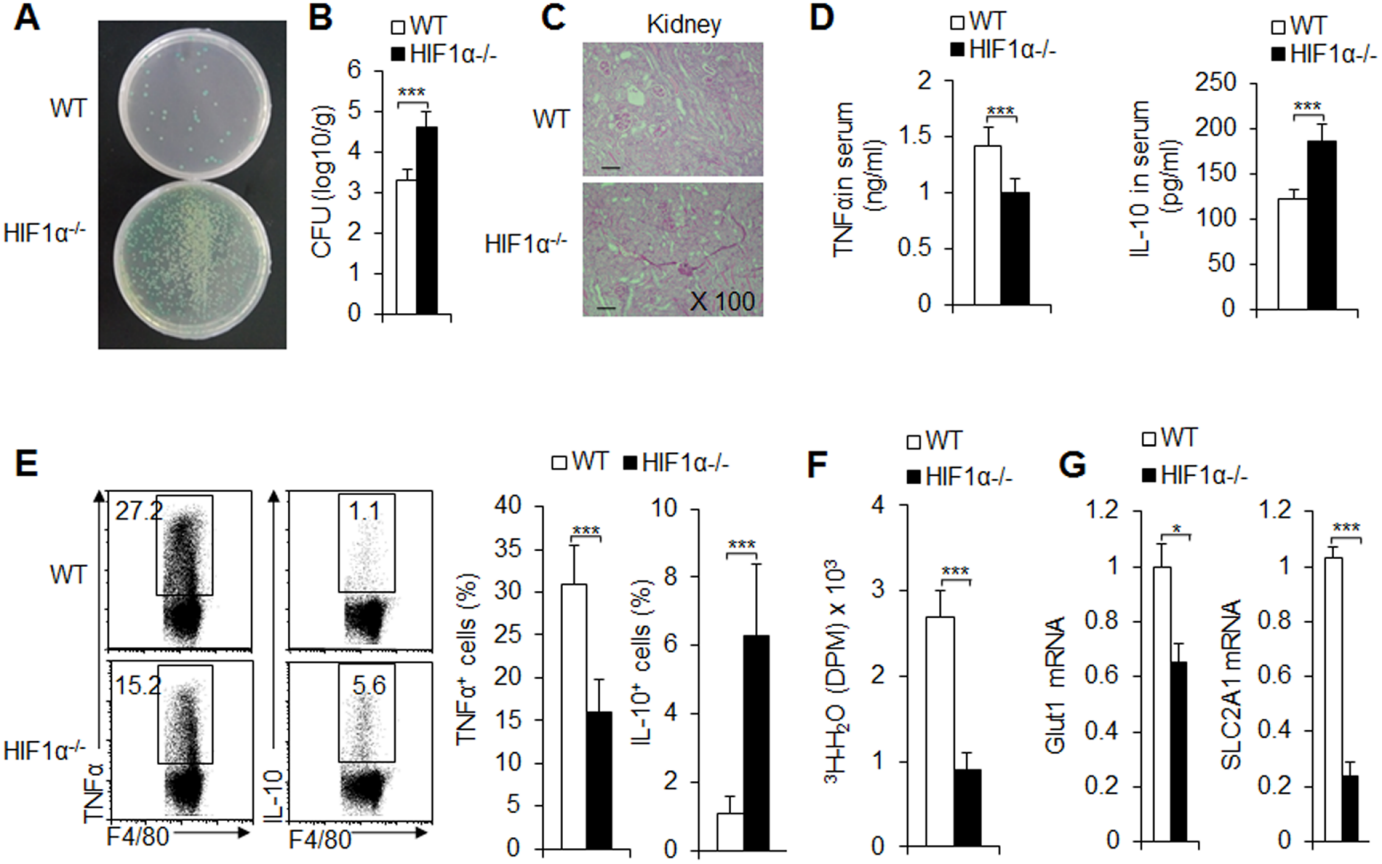
HIF1α is required for pro-inflammatory macrophage differentiation following *C. albicans* fungal infection. C57BL/6 WT or HIF1α^−/−^ mice were i.v. injected with 2 × 10^5^ of live *C. albicans* yeast. After 9 days, mice were killed for analysis. Infected kidneys were collected and the fungal burden in the kidneys is shown (**A**), expressed as CFU per g (**B**), and an image of the H&E staining of pathological kidney injuries is shown (**C**); serum was collected, and the indicated cytokine was determined using an ELISA (**D**). At the same time point during infection, the PEMs were collected, and the indicated cytokine expression was analyzed with FCM; a representative figure is shown in the left image, and data are summarized in the right image (**E**). The glycolytic pathway activity of PEMs was also determined (**F**,**G**). Data are presented as the means ± SD (n = 3–5). One representative experiment of three independent experiments is shown. *P < 0.05 and ***P < 0.001, compared with the indicated groups.

We challenged WT and HIF1α^−/−^ mice with *C. albicans in vivo* and compared their effects with those of *L. monocytogenes* infection. HIF1α deficiency significantly increased the survival of the fungus (Fig. 4A,B) and revealed a more severe alteration in histopathological inflammation (Fig. 4C). After *C. albicans* challenge and testing in the serum of mice and the supernatant of cultures from peritoneal macrophages, we consistently observed that HIF1α deficiency significantly decreased the levels and expression of the pro-inflammatory cytokine TNFα and increased the level and expression of the anti-inflammatory cytokine IL-10 (Figs 4D,E and S8A,B). The HIF1α^−/−^ peritoneal macrophages showed significantly decreased glycolytic pathway activity (Figs 4F,G and S8C). Additionally, NO production and iNOS expression, both markers of pro-inflammatory macrophages, were also expressed at lower levels in HIF1α deficient macrophages (Fig. S9). Together, these data showed that HIF1α is required for macrophage differentiation during fungal infection.

### Glycolytic activity is involved in the HIF1α-directed macrophage differentiation

**Figure 5:**
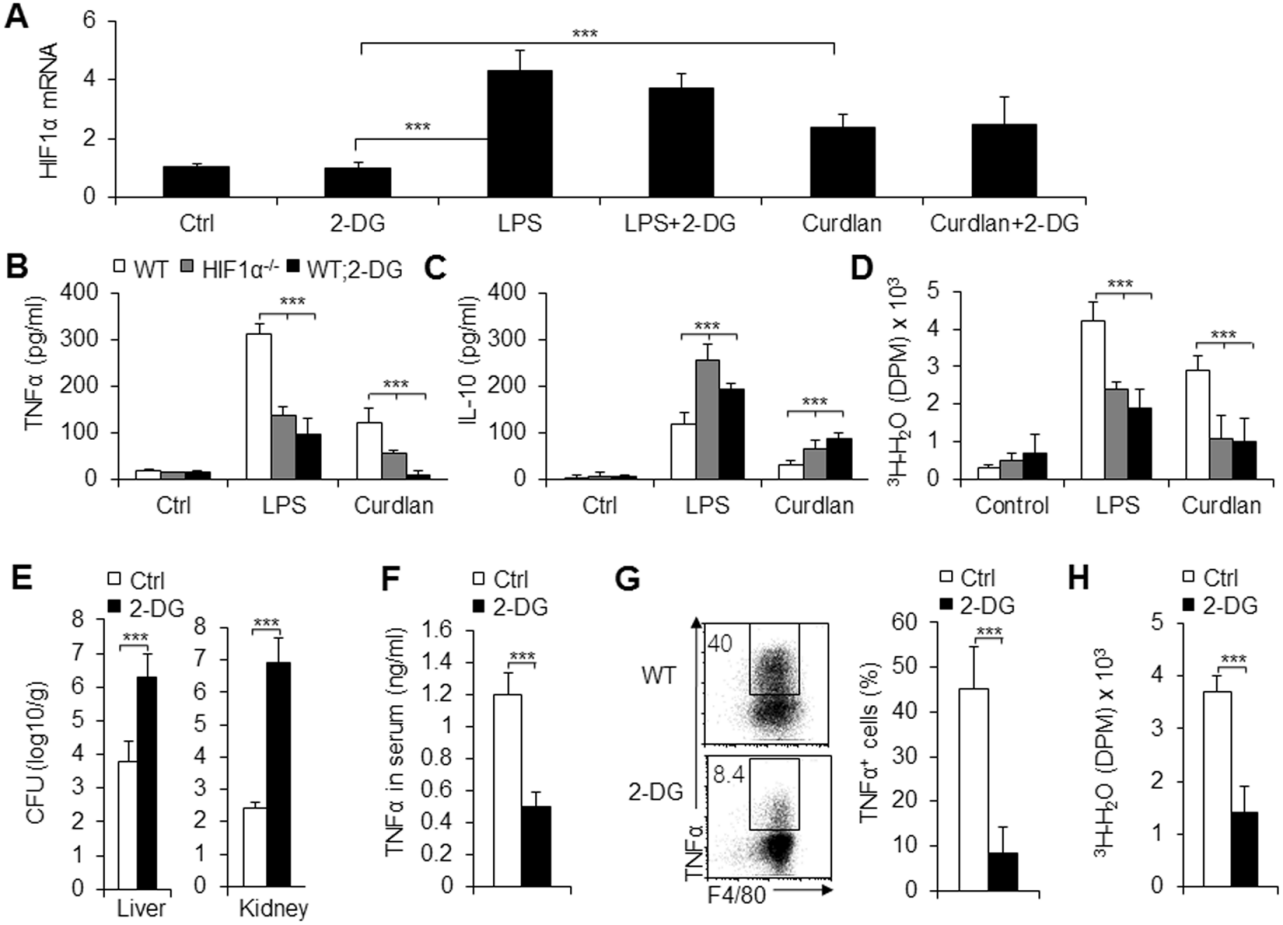
Glycolytic pathway activity was required for pro-inflammatory macrophage differentiation. PEMs with indicated treatments for 10–12 h (2-DG, 1 mmol/L; LPS, 100 ng/mL; curdlan, 100 ng/mL; **A**–**D**). **A**. HIF1α mRNA expression of PEMs was determined with qPCR (Value of control groups was set to 1). (**B** and **C)** Supernatants were collected, and the indicated cytokine concentration was determined using an ELISA. (**D**) The glycolytic pathway activity was summarized. Mice were i.p. injected with 1 × 10^5^ of *C. albicans* yeast and also injected intraperitoneally with 2-DG (2 g/kg body weight) or solvent alone (PBS; Ctrl) for 9 days. 2-DG or PBS were given daily up until the day before the mice were euthanized (**E**–**H**). (**E**) Mouse liver and kidneys were collected, and the CFU was evaluated. (**F**) Serum TNFα concentration (**F**). (**G**) TNFα expression of PEMs with FCM. A representative image is shown in the left figure and data are summarized in the right figure. (**H)** The glycolytic pathway activity of PEMs. Data are presented as the means ± SD (n = 3–4). One representative experiment of three independent experiments is shown. *P* < 0.001, compared with the indicated groups.

To investigate whether glycolytic activity is involved in the HIF1α-directed macrophage differentiation during different pathogenic microorganism infections, we cultured and stimulated peritoneal macrophages from HIF1α^−/−^, WT, or 2-DG pretreated WT (WT; 2-DG) mice with either LPS or curdlan. LPS and curdlan significantly increased the mRNA expression of HIF1α, while blocking glycolytic pathway activity with 2-DG, a prototypical inhibitor of the glycolytic pathway, by blocking hexokinase, the first rate-limiting enzyme of glycolysis, did not alter the expression of HIF1α (Fig. 5A). However, blocking glycolytic pathway activity with 2-DG treatment significantly inhibited the differentiation of pro-inflammatory macrophages after stimulation with LPS or curdlan in WT macrophages or with different pathogenic microorganism infections. This is consistent with HIF1α deficiency-induced macrophage phenotypic changes (Fig. 5B–D). Furthermore, blocking glycolytic pathway activity with 2-DG treatment *in vivo* significantly decreased the secretion and expression of the pro-inflammatory cytokine TNFα, and increased the fungus’ survival (Fig. 5E–H). These data suggested that glycolytic pathway activity is involved in the HIF1α-directed macrophage differentiation during different pathogenic microorganism infections.

### Pharmacological targeting of HIF1α-dependent glycolytic pathway activity in mouse and human macrophage differentiation

**Figure 6:**
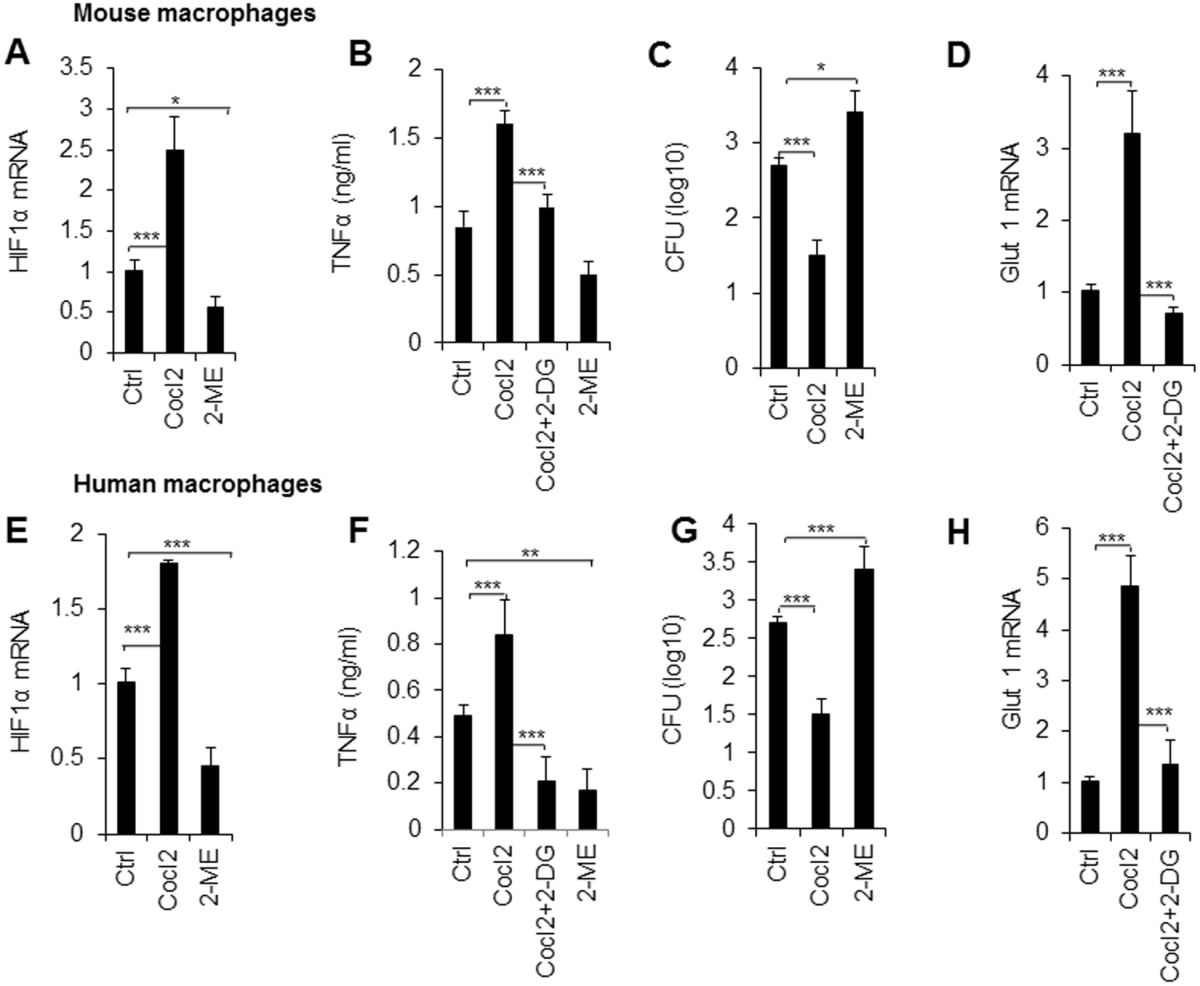
Pharmacologically targeting HIF1α and glycolytic pathway activity in mouse and human cells. Mouse PEMs (**A**–**E**) or human macrophages (**F**–**J**) pulsed with *C. albicans* yeast (1 × 10^5^) for 3 days in the absence or presence of CoCl_2_ (200 µM), 2-ME (2 µM), or 2-DG (1 mmol/L). (**A** and **E)** The HIF1α mRNA expression of macrophages was determined with qPCR. (**B** and **F**) The TNFα concentration of supernatants was determined. (**C** and **G**) The CFU was evaluated. (**D** and **H)** The Glut1 mRNA expression was determined with qPCR. Data are presented as the means ± SD (n = 3–4). One representative experiment of three independent experiments is shown. *P < 0.05 and ***P < 0.001, compared with the indicated groups.

Next, we applied a pharmacological approach to target the HIF1α-glycolytic pathway in both mouse and human macrophages to determine whether we can recapitulate our findings from the infectious inflammation model. Treatment with CoCl_2_, an activator of HIF1α, upregulated HIF1α expression, TNFα secretion, and fungal death. Treatment with 2-ME, an inhibitor of HIF1α, significantly downregulated HIF1α expression, TNFα secretion, and increased fungal survival in mouse pro-inflammatory macrophages (Fig. 6A–C). Blocking glycolytic pathway activity significantly inhibited the secretion of TNFα caused by HIF1α upregulation (Fig. 6B and D). We extended our experiments to the differentiation of human pro-inflammatory macrophages, and largely recapitulated what we observed in genetic and pharmacological targeting of the differentiation of mouse pro-inflammatory macrophages in terms of the alteration in fungal survival, the secretion of the pro-inflammatory cytokine TNFα, and glycolytic pathway activity (Fig. 6E–H). Thus, our data indicated that HIF1α-dependent glycolytic pathway activity mediated an evolutionarily conserved signaling pathway in both mouse and human macrophages during infectious inflammation.

## Discussion

Host defenses against infection rely on innate immune cells that sense microbe-derived products through pattern recognition receptors, such as toll-like receptors (TLRs) and c-type lectins, NOD-like receptors, RIG-I-like receptors and cytosolic DNA sensors^36,37^. Macrophages are important first-line innate immune defense cells that upon encountering microbial ligands from bacterial or fungal microorganisms could differentiate into differently polarized cells and cause a series of innate immune defense responses^38^. Previously, our study^19^ showed that HIF1α is critical for the differentiation of pro-inflammatory macrophages; however, its significance remains unclear during pathogenic infections, especially since different pathogenic microorganisms cause different macrophage polarization through specific signaling mechanisms. To systemically clarify the effects of HIF1α in the polarization of pro-inflammatory macrophages during different pathogenic microorganism infections, we selected two kinds of pathogens, *L. monocytogenes*, a classical gram-positive bacterium, and *C. albicans*, a classical fungal organism, and observed the alteration of macrophage polarization following *in vitro* and *in vivo* challenge. Additionally, we examined the role of HIF1α-dependent glycolytic pathway activity on cellular polarization. The results showed that bacterial and fungal infections exhibited similar effects on macrophage phenotypic differentiation. HIF1α deficiency inhibits pro-inflammatory macrophage functional differentiation when cells are stimulated with LPS or curdlan *in vitro* or when mice are infected with *L. monocytogenes* or *C. albicans in vivo*, leading to a decreased secretion of the pro-inflammatory cytokines TNFα and IL-6, secretions associated with pathogenic microorganism survival. Increased survival of pathogenic microorganisms caused a much more severe infectious inflammation. Alteration of glycolytic pathway activation was required for the differentiation of pro-inflammatory macrophages in protecting against bacterial and fungal infections. However, which specific macrophage cell subtype is mainly affected by HIF1α still needs to be investigated. HIF1α deficiency significantly increases the production of IL-10 protein by macrophages following infection but not IL-10 mRNA expression, indicating that IL-10 secretion is regulated by HIF1α through post-transcriptional regulatory mechanisms. Additionally, in the present study, LysM-Cre was used to delete the HIF1α in myeloid cells, at least including macrophages and neutrophils. Therefore, the effect of infection in mice does not fully represent the effect of macrophage *in vitro*. It has been confirmed that HIF1α is critical in regulating neutrophil survival and recruitment in inflammation^39,40^. So, the effect of neutrophil and other myeloid cell elimination should also be taken into account in the mouse disease model. Thus, combined with the experimental results *in vivo* and *in vitro*, the HIF1α-dependent glycolytic pathway is essential for the macrophage functional differentiation in protecting against bacterial and fungal infections (Fig. S10).

Macrophages, in protecting against pathogenic organism infections, have a unique plasticity that allows them to differentiate into differently polarized phenotypes depending on the cytokine microenvironment^41,42^. Pro-inflammatory macrophages are differentiated by type I pro-inflammatory cytokines and microbial products, such as LPS, and express most TLRs, opsonin receptors, inducible NO synthase (iNOS), and secrete the cytokines TNFα and IL-6, among others. In contrast, anti-inflammatory macrophages are differentiated by type II anti-inflammatory cytokines (IL-4 and/or IL-13) and ameliorate type I inflammation^43^. Fungal pathogens, such as *C. neoformans*, *H. capsulatum*, *P. brasiliensis*, *Coccidioides immitis*, and *C. albicans* are sensitive to the antimicrobial effects of macrophages. However, many fungi use various mechanisms to interact with macrophages and cause different macrophage inflammatory responses^44–46^. In the case of *H. capsulatum*, macrophages can efficiently phagocytize the fungus but are unable to kill it until activated by cytokines produced by effector T cells^47^. *C. albicans* and *C. neoformans* are also thought to mediate NO suppression through the downregulation of macrophage iNOS RNA and usually inhibit pro-inflammatory macrophage phenotypes^44^. Thus, macrophage polarization effects need to be explored for their role in protecting different pathogenic microorganisms during infection. Here, we showed that *C. albicans* can cause significant pro-inflammatory macrophage polarization and that HIF1α-glycolytic pathway mechanisms are required for the mediation of pro-inflammatory macrophage polarization in protecting against *C. albicans* fungal infection *in vivo* and *in vitro*.

Macrophage biology in humans is less understood compared to that in animal model organisms because of the technical and ethical challenges in obtaining fresh material from human subjects. While several protocols that could differentiate blood monocytes into macrophages (monocyte-derived macrophages) by applying GM-CSF or M-CSF have been available since the 1990s^48,49^, the difficulties of accessing human peripheral tissue leukocytes, including resident macrophages, remains a technical barrier for studying human macrophage biology. To date, most studies have relied on the use of human monocyte-derived macrophages. These *in vitro* models have allowed us to gain enormous insights into the functions of these cells, but the physiological relevance of some key aspects is still unclear^50–52^. Recent comparative biology studies have revealed that certain human and mouse macrophage subsets share some functional and phenotypical characterizations and therefore may represent homologous subsets^51–54^. For instance, human CD14^+^ classical monocytes are considered as a homologous subset to murine Ly6C^hi^ monocytes, since M-CSF stimulated human monocyte-derived macrophages displayed characteristics of alternatively activated macrophage (termed M2-like) phenotypes^51,55–57^. In recent years, this has replaced the simplistic view of myeloid precursors giving rise to blood monocytes that, in turn, originate tissue macrophages^58,59^. Similar to human peritoneal macrophages, mouse peritoneal macrophages (F4/80^hi^, CD11b^hi^, and MHCII^lo^) are believed to derive from an embryonic precursor^60–62^. In the current studies, we have shown that fungal infection drives up glycolysis and promotes pro-inflammatory cytokine production in both human monocyte-derived macrophages and mouse peritoneal macrophages. Importantly, these effects are mediated through the transcription factor HIF1α. Furthermore, our findings showed that pharmacologically targeting HIF1α could direct mouse and human macrophage differentiation in a similar manner. Our studies implicate metabolic programs as promising therapeutic targets for macrophage-driven human diseases.

Although it is known that glycolysis is associated with innate immune cell-mediated inflammation, as well as playing a role in immune-associated diseases, the mechanisms regulating increased glycolysis following pattern recognition receptor (PRR) stimulation particularly in macrophage polarization are unclear, especially in *in vivo* disease models. HIF1α plays a crucial role in the regulating myeloid cell function in hypoxia and in inflammation more broadly. Conditional knockout mice lacking HIF1α in neutrophils, macrophages and dendritic cells were demonstrated to have profoundly impacted immune functions. Using myeloid-targeted HIF1α knockout mice, Cramer *et al*. have confirmed a critical role for HIF1α in regulating neutrophil and mononuclear cell phagocytosis^63^. In HIF1α-deficient myeloid cells, this resulted in a reduction of ATP pools, accompanied by profound impairment of bacterial killing. *In vivo* this correlated with the ablation of ablation of sodium dodecyl sulphate-induced cutaneous inflammation and a reduction in synovial infiltration in an immune complex-mediated inflammatory arthritis model. There are follow up studies that demonstrated that HIF1α promotes myeloid cells (macrophages and/or neutrophils) pro-inflammatory activation and antimicrobial defense in a streptococcal infection model^64^. It showed that HIF1α deficient in myeloid cells decreased the bactericidal activity and exaggerated systemic spread of infection. In the present study, we showed that *L. monocytogenes*, a classical gram-positive bacterium infection directed a pro-inflammatory macrophage differentiation *in vitro* and *in vivo*. Moreover, it has been demonstrated that HIF1α in myeloid cells plays a role in trained immunity and defense against *C. albicans* fungal infection^32^. Trained monocytes display high glucose consumption, lactate production, and NAD^+^/NADH ratio, reflecting a shift in the metabolism of trained monocytes with an increase in glycolysis dependent on the activation of mTOR through HIF1α pathway. Inhibition of mTOR, or HIF1α blocked monocyte induction of trained immunity. Mice with a myeloid cell-specific defect in HIF1α were unable to mount trained immunity against bacterial sepsis. Otherwise, there are data that demonstrated that HIF1α plays an important role in antifungal responses^65,66^. Our previous study^19^ showed that pro-inflammatory stimulation suppresses Myc-dependent cell proliferation while engaging a HIF1α-dependent transcriptional program to maintain heightened glycolysis in pro-inflammatory macrophage functional differentiation. However, whether HIF1α-dependent glycolysis is critical for macrophage polarization during *in vivo* infection with different pathogenic microorganisms remains uninvestigated. In the present study, we further showed that HIF1α-dependent glycolytic pathway is essential for the macrophage functional differentiation in protecting against bacterial and fungal infections *in vitro* and *in vivo*.

## Electronic supplementary material

Supplementary Figure
