# Supplementary material for: HIF1α-dependent glycolysis promotes macrophage functional activities in protecting against bacterial and fungal infection
Source: Sci Rep. 2018 Feb 26;8:3603. doi: 10.1038/s41598-018-22039-9 (PMC5827022; doi:10.1038/s41598-018-22039-9)
Supplement: Supplementary file 1 — Supplementary Figure [file 41598_2018_22039_MOESM1_ESM.pdf]

HIF1α-dependent glycolysis promotes macrophage functional activities in protecting against bacterial and fungal infection

Chunxiao Li[1,2,#], Yu Wang[1,2,#], Yan Li[1,2,#], Qing Yu[2,#], Xi Jin[2], Xiao Wang[1,2], Anna Jia[2], Ying Hu[2], Linian Han[2], Jian Wang[1,2], Hui Yang[1], Dapeng Yan[1,*], Yujing Bi[3,*], Guangwei Liu[1,2,*]

[1]Department of Immunology, School of Basic Medical Sciences, Fudan University, Shanghai 200032 China; [2]Key Laboratory of Cell Proliferation and Regulation Biology of Ministry of Education, Institute of Cell Biology, College of Life Sciences, Beijing Normal University, Beijing 100875 China; [3]State Key Laboratory of Pathogen and Biosecurity, Beijing Institute of Microbiology and Epidemiology, Beijing 100071, China

Fig. S1

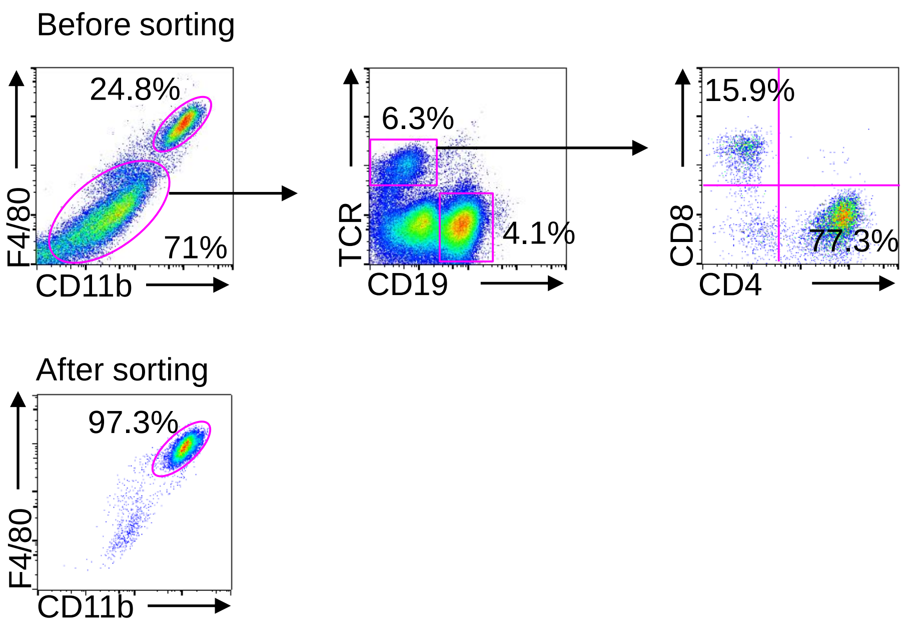

Fig. S1. Sorting and confirmation of peritoneal exudate macrophages.
Peritoneal exudate macrophages (PEMs) were sorted and confirmed with flow cytometry and typical figure showed before sorting and after sorting.

Fig. S2

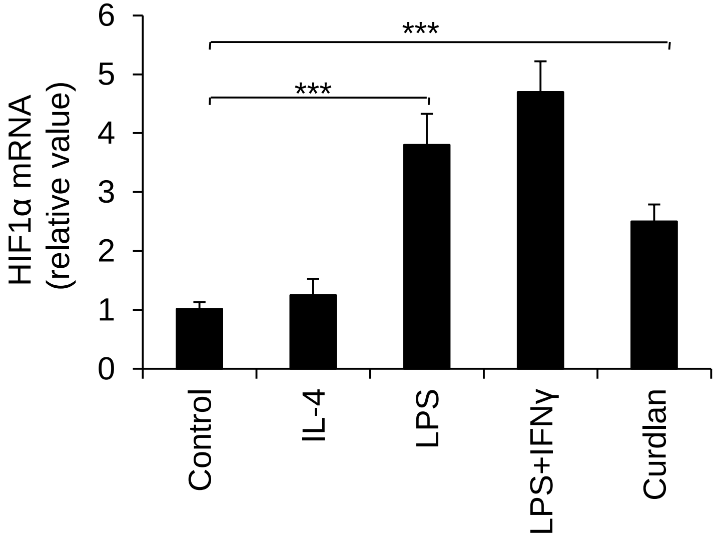

Fig. S2. HIF1α-dependent glycolysis is associated with pro-inflammatory macrophage differentiation during inflammation
Sorted peritoneal exudate macrophages (PEMs) from C57BL/6 mice were stimulated with IL-4 (1000 U), LPS (100 ng/mL), LPS + IFNγ (100 ng/mL) or curdlan (100 ng/mL) for 10-12 h, the HIF1α mRNA expression was determined using qPCR (value of control groups was set to 1). Data is presented as the means±SD (n=4). One representative experiment of three to four independent experiments is shown. ***, $P<0.001$, compared with the indicated groups.

Fig. S3

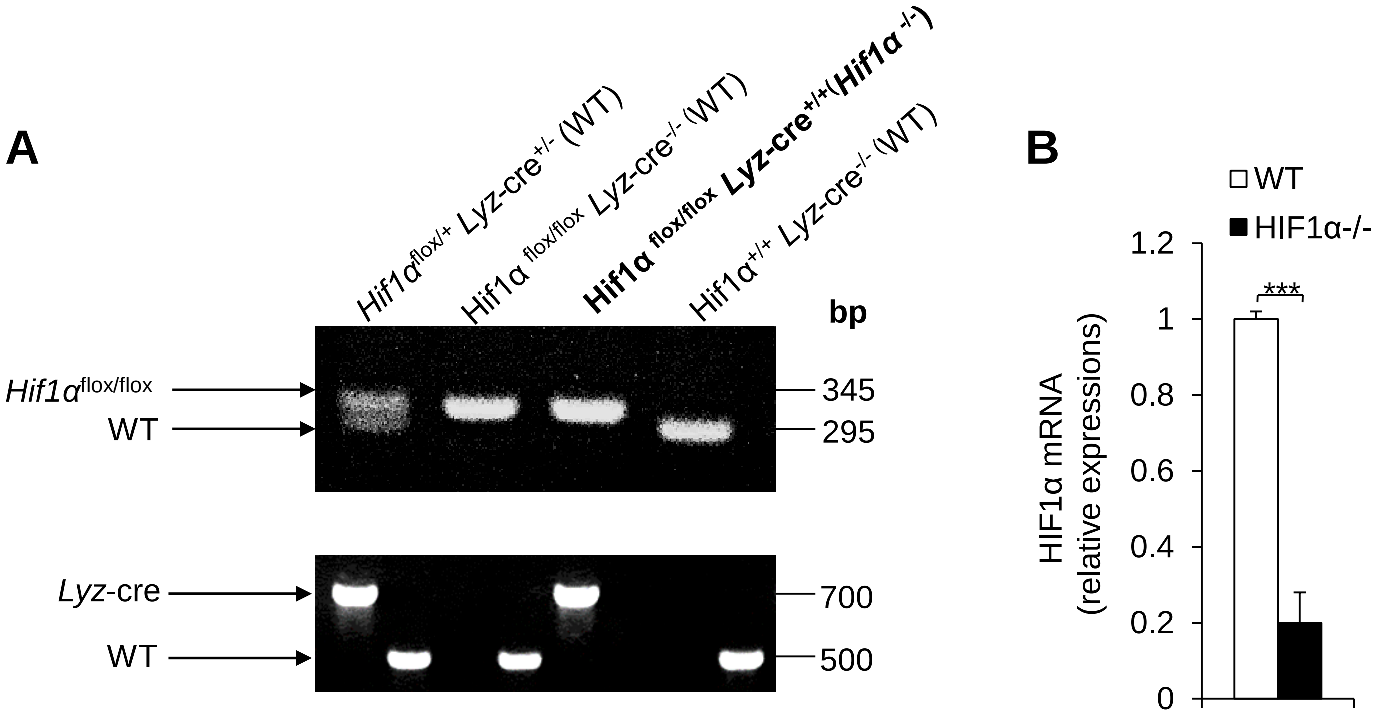

Fig. S3. Generation and confirmation of myeloid cell-specific HIF1α deficient mice.

A. Representative genotyping PCR of *Hif1α*$^{-/-}$ and wild-type (WT) mice. **B**. mRNA expression of HIF1α in peritoneal exudate macrophages (PEMs) isolated from *Hif1α*$^{-/-}$ and WT mice. Data have been presented as mean ± s.d. (n=3-4). Results are representative of three independent experiments. ***, *P*<0.001, compared with the indicated groups.

Fig. S4

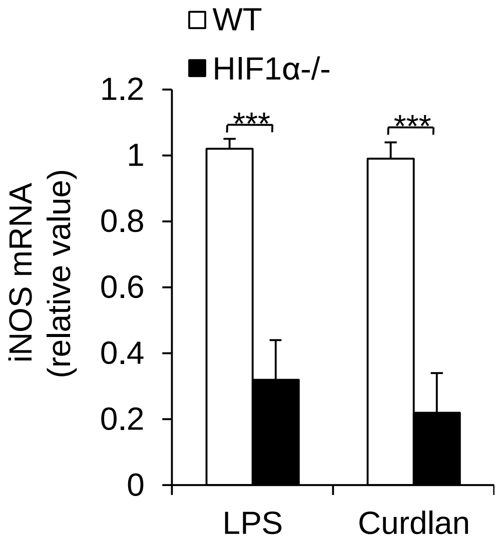

Fig. S4. HIF1α is critical for pro-inflammatory macrophage differentiation *in vitro* .
Sorted F4/80^{+} peritoneal exudates macrophages from WT and HIF1α$^{-/-}$ mice with stimulated with LPS (100 ng/mL) or Curdlan (100 ng/mL) for 10-12 h and the iNOS mRNA expression were determined with qPCR. Data have been presented as mean±s.d. (n=3). Results are representative of three independent experiments. ***, P<0.001, compared with the indicated groups.

Fig. S5

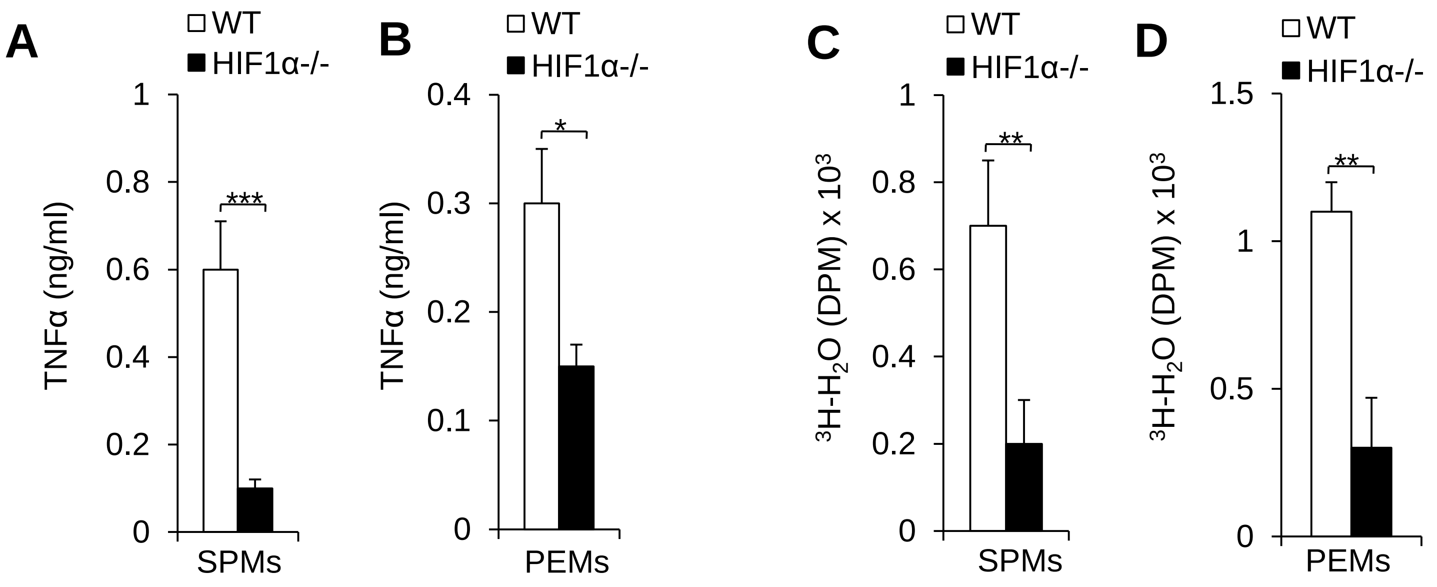

Fig. S5. HIF1α is required for pro-inflammatory macrophage differentiation following *Listeria* bacterial infection.
C57BL/6 WT or HIF1α$^{-/-}$ mice were i.v. injection 1 x 10^5 *L. monocytogenes* bacteria. At the 48 h after infection, the sorted splenic macrophages (SPMs) or PEMs were cultured for 12 h, the supernatant was collected and TNFα were determined with Elisa (**A**&**B**) and the glycolytic activities of macrophages were determined (**C**&**D**). Data have been presented as mean±s.d. (n=3). Results are representative of three independent experiments. *, *P*<0.05, **, *P*<0.01 and ***, *P*<0.001, compared with the indicated groups.

Fig. S6

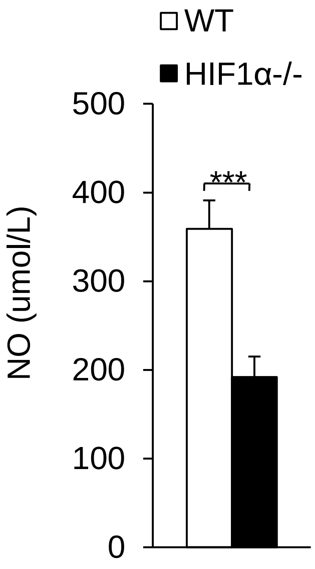

Fig. S6. HIF1α is required for pro-inflammatory macrophage differentiation following *Listeria* bacterial infection.
C57BL/6 WT or HIF1α$^{-/-}$ mice were i.v. injection 1 x 10^5 *Listeria* bacteria. At the 48 h after infection, the mouse F4/80$^+$ peritoneal exudates macrophages were sorted and stimulated with LPS for 12 h, the supernatant was collected and NO production were determined with Griess reagent. Data have been presented as mean±s.d. (n=3). Results are representative of three independent experiments. ***, P<0.001, compared with the indicated groups.

Fig. S7

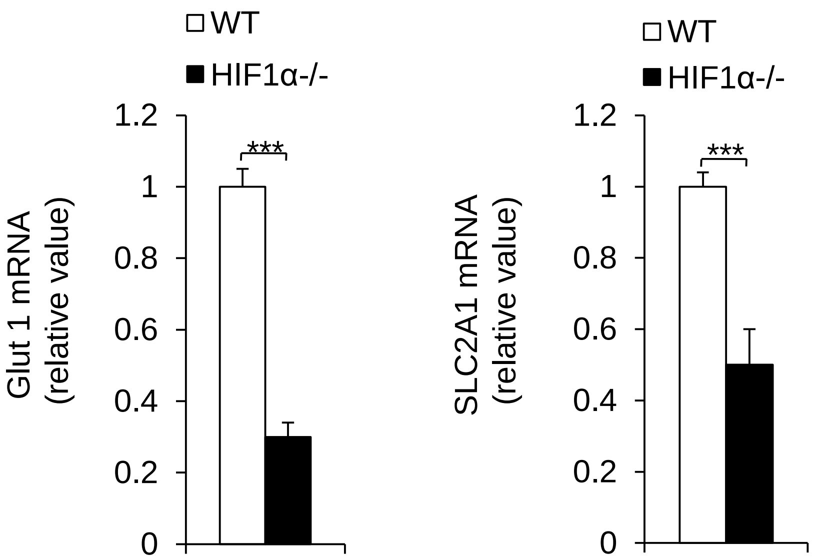

Fig. S7. HIF1α-dependent glycolytic activities are required for pro-inflammatory macrophage differentiation following *Listeria* bacterial infection.

C57BL/6 WT or HIF1α$^{-/-}$ mice were i.v. injection 1 x 10^5 *Listeria* bacteria. At the 48 h after infection, the sorted mouse PEMs were stimulated with LPS for 12 h, the glycolytic activity molecules of macrophages were determined with qPCR. Data have been presented as mean$\pm$s.d. (n=3-5). Results are representative of three independent experiments. ***, *P*<0.001, compared with the indicated groups.

Fig. S8

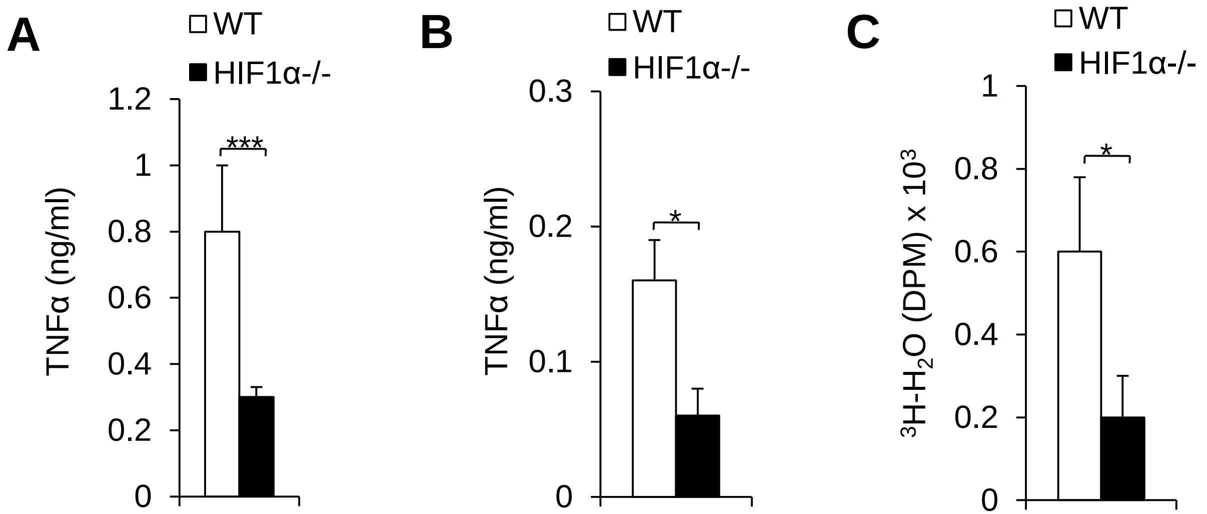

Fig. S8. HIF1α is required for pro-inflammatory macrophage differentiation following *C.albians* fungal infection.

C57BL/6 WT or HIF1α$^{-/-}$ mice were i.v. injection 2 x 10^5 live *C. albians* yeast. After 9 days, the sorted PEMs were cultured with (**A**) or without (**B**) LPS for 12 h, the supernatant was collected and TNFα were determined with Elisa and the glycolytic activities of macrophages were determined (**C)**. Data have been presented as mean±s.d. (n=3). One representative of three independent experiments has been shown. *, *P*<0.05 and *P*<0.001, compared with the indicated groups.

Fig. S9

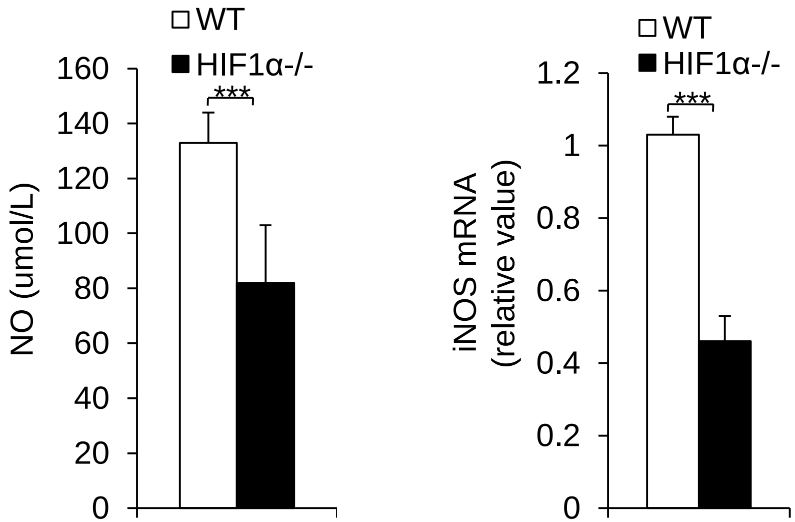

Fig. S9. HIF1α is required for pro-inflammatory macrophage differentiation following *C.albians* fungal infection

C57BL/6 WT or HIF1α$^{-/-}$ mice were i.p. injection 2 x 10^5 live *C.albians* yeast. After 9 days, mice were killed for analysis. Infected mouse F4/80$^+$ macrophages were sorted and stimulated by *C.albians* yeast for 48 h and supernatant were collected and NO productions were determined with Griess reagent (**A**). The iNOS mRNA relative expression of F4/80$^+$ macrophages were determined with qPCR (**B**). Data have been presented as mean±s.d. (n=3). Results are representative of three independent experiments. ***, *P*<0.001, compared with the indicated groups.

Fig. S10

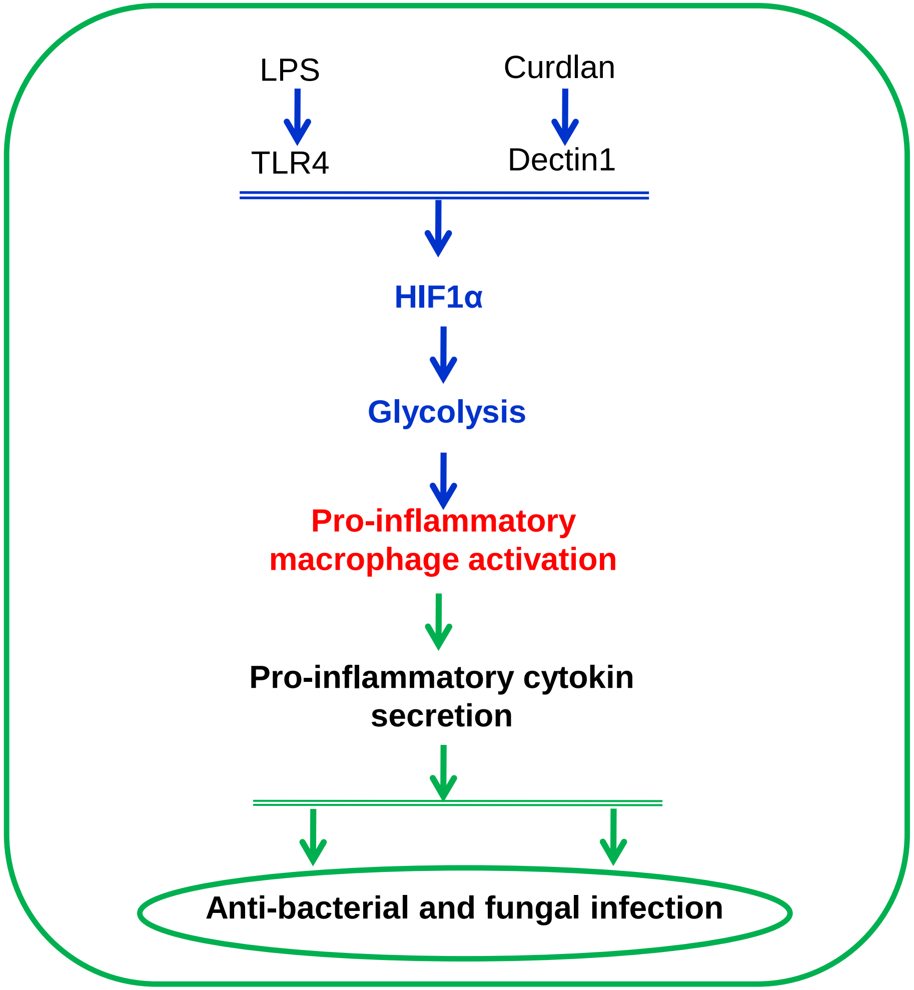

Fig. S10 Proposed model of how HIF1α in macrophages integrates innate stimuli to control pro-inflammatory macrophage differentiation in protecting against bacterial and fungal pathogen infection.
